# Skin infection boosts memory B-cells specific for a cryptic vaccine epitope of group A streptococcus and broadens the immune response to enhance vaccine efficacy

**DOI:** 10.1038/s41541-018-0053-9

**Published:** 2018-04-26

**Authors:** Manisha Pandey, Victoria Ozberk, Emma L. Langshaw, Ainslie Calcutt, Jessica Powell, Michael R. Batzloff, Tania Rivera-Hernandez, Michael F. Good

**Affiliations:** 1 Institute for Glycomics, Gold Coast Campus, Griffith University, Queensland, Australia; 2 School of Chemistry and Molecular Biosciences, University of Queensland, Brisbane, Australia

## Abstract

Antigenic diversity of the M protein is a major constraint to the development of immunity to group A streptococcus (GAS). We demonstrate that a conserved cryptic epitope that is unrecognized by the host immune system following infection can protect mice following vaccination, and that immunity is strengthened and broadened following successive infections. The observation that infection can boost and broaden, but cannot prime immunity to a cryptic epitope, may be exploited for vaccines for other pathogens.

## Introduction

The immune system evolved to provide life-long protection from repeated infections; however, induction of immunity to group A streptococcus (GAS) is very inefficient, taking up to 20 years to develop.^1^ Consequently, this organism is responsible for significant morbidity and mortality, particularly among Indigenous and impoverished communities.^2^ Streptococcal pyoderma is responsible for invasive GAS disease and may be responsible for Australia’s Indigenous populations suffering the highest rates of rheumatic heart disease worldwide.^3^ Other Indigenous populations such as New Zealand Maoris, South Pacific Islanders, and First Nations people of Canada also suffer very high rates of streptococcal skin disease.^4,5^ Globally, GAS is responsible for the loss of over 500,000 lives per year.^2,6^ A vaccine is urgently needed.

The immunopathogenesis underlying the slow acquisition of immunity is not understood, but attributed to specific virulence factors impeding innate immunity and significant antigenic diversity of the type-specific M protein.^7,8^ Recently, we demonstrated that GAS infection of the skin leads to B-cell responses to serotypic M-protein determinants and strain-specific protective immunity; however, these are rapidly lost.^9^ Nevertheless, if re-infected with the same strain, persisting immunity and memory B-cells (MBCs) develop to type-specific epitopes and these are able to adoptively transfer strain-specific protection to naïve recipients.^9^ Thus, unless re-infected with the same strain, long-lasting protective MBCs may never develop to that strain, which, together with the enormous diversity of the M protein, contributes to streptococcal endemicity in at-risk populations. Furthermore, even with repeated infections of mice, antibodies do not develop to a conserved Mprotein epitope, defined by “J8”,^10^ that by itself is able to induce strong immunity following vaccination.^9,11,12^ This “cryptic” epitope is essentially invisible to antigen-inexperienced B-cells following infection. While J8-vaccine-induced antibodies can protect against multiple strains, these observations raised questions as to whether J8-specific MBCs would persist and be boosted following infection or whether the epitope would also remain invisible to the MBCs leading them to diminish over time with loss of immunity.

Here, we asked if immunity induced by J8 conjugated to diphtheria toxoid (J8-DT) would persist following infection. We further asked whether sequential infections would broaden and strengthen the immune response of vaccinated mice by enlisting protective B-cells of other specificities. Various other streptococcal proteins and sugars have been identified as the targets of protective antibodies and some are considered as vaccine candidates.^13^ One candidate is streptococcal cell envelope protease (SpyCEP), which is a CXC chemokine-cleaving protease that is upregulated in hyper-virulent organisms as a result of mutations within the two-component negative transduction system, *covR/S*. SpyCEP blocks the chemotaxis of neutrophils to the site of infection. As a result, such organisms are resistant to J8-vaccine-mediated protection. However, J8-mediated protection is restored if an immunogenic fragment of SpyCEP is included in the vaccine.^12,14,15^

We demonstrate here that not only do J8-specific MBCs persist following infection of J8-vaccinated mice, but that they are significantly boosted. Furthermore, the immune response of J8-vaccinated mice is broadened as a result of infection to include SpyCEP-specific B-cells, which renders J8-vaccinated mice now resistant to a hyper-virulent *covR/S* mutant strain. This mechanism of “infection-mediated vaccine enhancement” (IMVE) may be relevant to other organisms that currently challenge vaccine development.

## Results and discussion

**Fig. 1:**
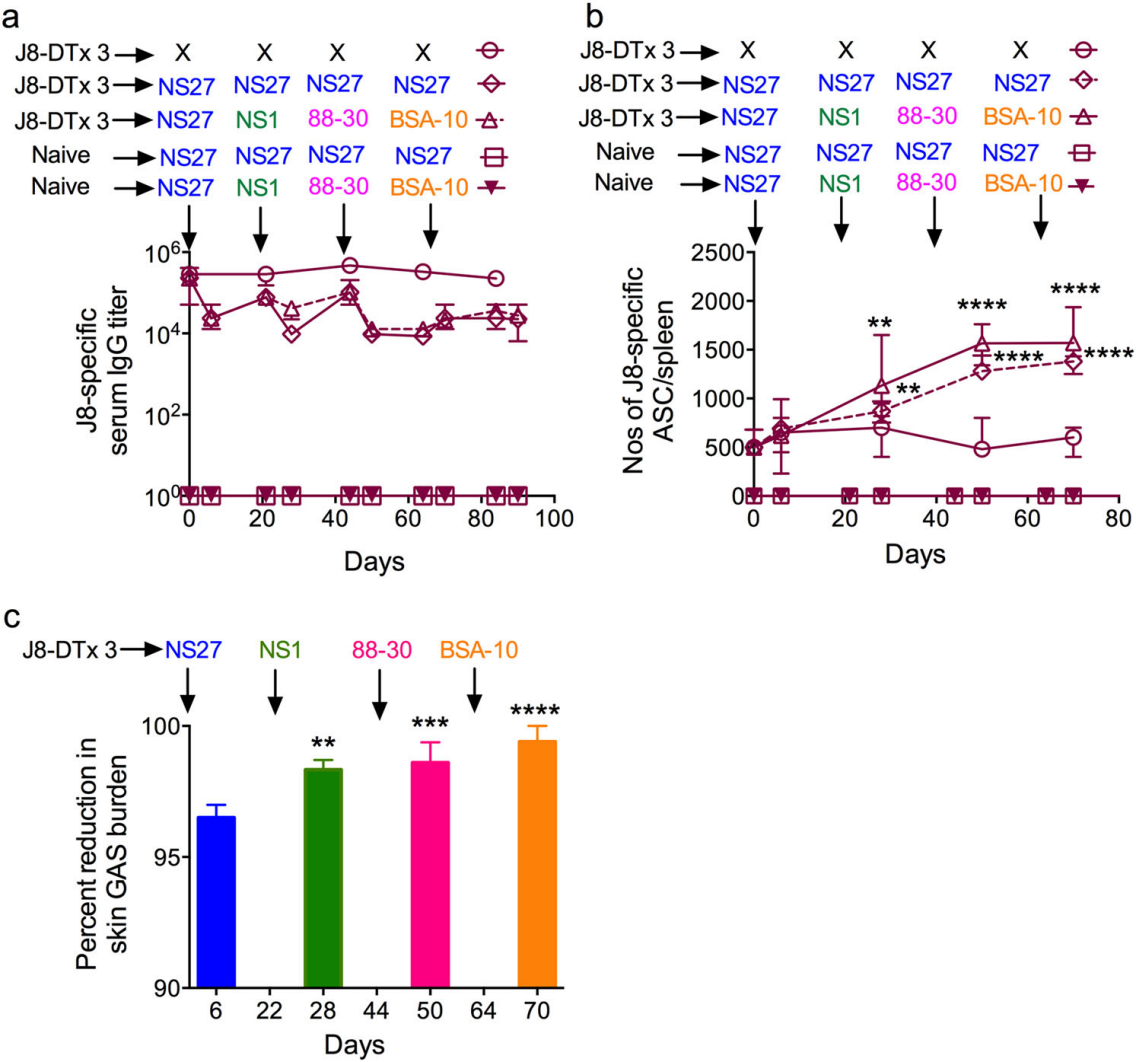
**a** Effect of J8-DT vaccination on immune responses following multiple sequential GAS infections. J8-DT-vaccinated (s.c. immunization x 3) or naïve BALB/c mice (*n* = 15/group) were sequentially infected with the same NS27 or four different GAS strains. Vaccinated-uninfected mice were also included. On days 6 and 21 post each infection, sera were collected and J8-specific IgG were measured via ELISA. Sera from the naïve-uninfected mice were used as a control in ELISA. Data are mean ± SEM. **b** Quantification of antibody secreting cells post vaccination and/or infection. Cohorts of J8-DT-vaccinated or naïve (*n* = 15/group) mice were sequentially infected multiple times with the same NS27 or four different GAS strains. To assess the development of memory, a designated number of mice were culled on day 6 post each infection and spleens were harvested. The numbers of ASCs specific for J8 were enumerated using ELISPOT. To assess the effect of multiple infections on boosting of ASC response, vaccinated-uninfected mice were used as controls. The data are mean ± SEM. Statistical analysis was performed using one-way ANOVA with Dunnett's multiple comparison test to compare numbers of ASCs post first infection with numbers of ASCs following each sequential infection. **p < 0.01 and ****p < 0.0001. **c** Protective efficacy of J8-DT following sequential infections with multiple GAS strains. Cohorts of BALB/c mice (*n* = 20–25/group) were immunized subcutaneously with J8-DT/Alum. Two weeks post last immunization mice were sequentially infected with four different GAS strains (NS27, NS1, 88-30, and BSA-10) via the skin. For each infecting GAS strain, naïve mice were also included as a challenge control. Each sequential infection was 3 weeks apart. On day 6 post infection, five mice each from immunized and control groups were sacrificed and skin samples collected to determine bacterial burden. Percent reduction in skin bacterial burden was calculated by taking into account the corresponding naïve challenge controls and is shown as mean ± SEM. The GAS burden (mean CFU) in control mice ranged between 747,800 and 915,280. Statistical analysis was performed using one-way ANOVA with Dunnett’s multiple comparison test to compare percent reduction in GAS burden post first infection with percent reduction in GAS burden following each sequential infection. **p < 0.01, ***p < 0.001, ****p < 0.0001

We previously showed that infection does not lead to the development of antibodies nor antibody secreting cells (ASCs) to J8.^9^ This raised doubt as to whether J8-vaccine-induced antibodies would be boosted as a result of new infections. We studied the responses of J8-vaccinated and non-vaccinated mice following sequential GAS infections. Again we observed that non-vaccinated mice did not generate antibodies to J8 following infection (Fig. 1a). Surprisingly, however, we observed that repeated infection of J8-DT-vaccinated mice with either NS27 GAS or four different *emm* type strains sequentially, while not increasing the J8-specific IgG titers (Fig. 1a), did lead to a significant increase in J8-specific splenic ASCs (Fig. 1b). Cryptic epitopes such a as J8 are not recognized following natural infection; however, such targets when presented out of context (such as a peptide), can induce antibodies that recognize the native antigen and destroy the organism.^16^ Our data suggest that this epitope is presented during infection. Priming and boosting mice with three immunizations of J8-DT resulted in J8-specific MBCs which underwent rapid expansion following infection. ASC boosting mirrored the significantly enhanced immunity in the skin observed following each sequential infection (Fig. 1c). By linear regression analysis we observed a significant trend between: (i) the number of infections and the percentage reduction in GAS bioburden following the next infection (*R*^2^ = 0.7844, *p* < 0.001); (ii) the number of GAS infections and the number of ASCs (*R*^2^ = 0.6896, *p* < 0.001); and (iii) the number of ASCs and percent reduction in GAS bioburden (*R*^2^ = 0.7942, *p* < 0.001) (Supplementary Fig. 1a–c). The failure of the J8-specific antibody titers to increase following infection of vaccinated mice is likely due to the titers already being very high and with some antibodies being removed by the ongoing infections, as we observed previously.^11^ Thus, while infection cannot induce a J8-specific immune response in naïve mice, it can boost an established memory response that significantly enhances the level of vaccine-induced protection over time.

We were particularly surprised that sequential infection of vaccinated mice would lead to protection against the hypervirulent *covR/S* mutant strain, BSA-10, which was the last strain in sequence used to challenge the mice (Fig. 1c). If the primary infection of J8-DT-vaccinated mice was BSA-10, we observed only limited protection^12^ (Supplementary Fig. 2a). Likewise, a single co-infection of J8-DT-vaccinated mice with multiple strains, including BSA10, resulted in only limited protection (49%) in skin to BSA-10 while offering >90% reduction in bacterial loads for NS27, NS1, and 88-30 (Supplementary Fig. 2b). To induce protection against hypervirulent *covR/S* mutant strains it is necessary to co-vaccinate mice with both J8-DT and a recombinant or synthetic fragment of SpyCEP.^12,14,15^ This has been shown for other *covR/S* mutant strains and here we show this for BSA-10 (Supplementary Fig. 2a).

**Fig. 2:**
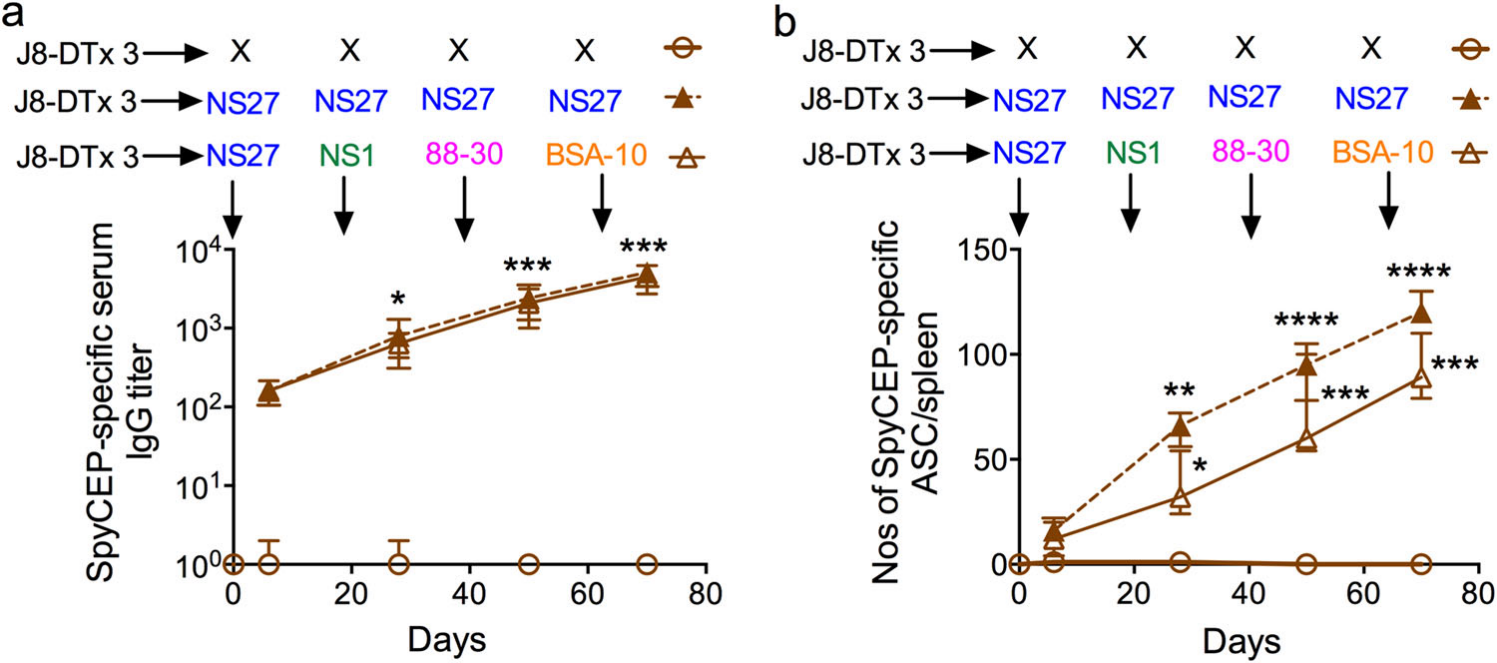
Induction of SpyCEP-specific responses in J8-DT-vaccinated mice following sequential infections with multiple GAS strains. J8-DT-vaccinated (s.c. immunization x 3) and naïve BALB/c mice (*n* = 15 mice/group) were sequentially infected multiple times with the same NS27 or four different GAS strains. Induction of SpyCEP-specific IgG (**a**) and ASC (**b**) was determined using ELISA or ELISPOT, respectively. J8-DT-vaccinated-uninfected mice were used as controls. Statistical analysis was performed using one-way ANOVA with Dunnett’s multiple comparison test to compare IgG titers or the numbers of ASCs post first infection with IgG titers or the numbers of ASCs following each sequential infection. *p < 0.05; **p < 0.01; ***p < 0.001; and ****p < 0.0001

We were curious as to whether sequential infections of J8-DT-vaccinated mice with multiple GAS strains had induced and boosted the immune response to SpyCEP, thus generating natural resistance to *covR/S* mutants in the presence of J8-specific antibodies. We observed that SpyCEP-specific antibodies and ASCs increased during the course of sequential skin infections of J8-DT-vaccinated mice commensurate with the increase in the level of protective immunity (Figs. 1c, 2a–b). The increased level of protection is significant and can be attributed to boosting of J8-specific memory and partially to the development of an immune response to SpyCEP during the course of the infections. Sequential infection of naïve mice with multiple GAS strains also induced similar levels of SpyCEP antibodies, but as these mice did not have J8-specific antibodies they did not develop immunity following a single infection (Supplementary Fig. 3a, c). Nevertheless, sequential infections with the same strain induced serotype-specific IgG and protection^12^ (Supplementary Fig. 3a, c).

Collectively, these data demonstrate that broadening of the host immune repertoire in vaccinated mice as a result of infection rendered the mice capable of resisting a subsequent infection with a highly virulent *covR/S* mutant strain that J8-DT alone could not accomplish. The enhanced protection correlated with a rise of antibodies and ASCs to SpyCEP, in keeping with the known synergistic requirement for both anti-J8 and anti-SpyCEP antibodies for protection against *covR/S* mutants.^12,14^ SpyCEP is highly conserved (>98% identity)^17^ and as such the broadening of the immune response as a result of infection has profound implications for vaccine coverage. However, SpyCEP-specific antibodies alone cannot provide protection to *covR/S* mutants, but only work in synergy with M protein-specific antibodies.^14,15^ Thus, sequential infection of naïve (unvaccinated) mice with different strains does not lead to protection. Our data do not exclude the likelihood that antibodies of other specificities arise as a result of multiple infections and that these also contribute to the enhanced level of protection.

These data have implications beyond streptococcus. A vaccine against a given strain of an organism may be sub-optimal but infection following vaccination may bring in immune responses to other specificities shared between different strains leading to enhanced protection. This model is consistent with a published study of a malaria vaccine in which vaccination alone gave minimal protection against infection but the level of protection increased with subsequent infections.^18^ This effect relied on the initial vaccination, as monkeys given an unrelated vaccine did not develop enhanced immunity following sequential infections.

In conclusion, we have shown that streptococcal infection boosts vaccine-induced immunity to a conserved cryptic epitope that is not recognized following infection of naïve mice. Furthermore infection broadens the immune repertoire resulting in protection against a hypervirulent *covR/S* mutant strain for which the vaccine alone does not protect and this correlates with an expansion in SpyCEP-specific ASCs and a rise in SpyCEP-specific antibodies. IMVE may be a general strategy for combatting other highly variable organisms.

## Methods

### Bacterial strains and generation of antibiotic resistance

All the GAS isolates were originally isolated from patients with skin infections in the Northern Territory of Australia. The strains were laboratory-adapted and grown on THB media.^9^ To allow for their selection during co-infection experiments, each strain was made resistant to a specific antibiotic as described previously.^9^

### Animals

All studies were approved by Griffith University’s Animal Ethics Committee in accordance with NHMRC guidelines. Specific pathogen-free 4–6-week-old female BALB/c mice were sourced from the Animal Resource Centre (Perth, Australia).

### Peptides

J8 (QAEDKVKQSREAKKQVEKALKQLEDKVQ) and K4S2 (KKKKNSDNIKENQFEDFDEDWENF) were synthesized and conjugated to DT as described.^14,15^ The recSpyCEP peptide was synthesized at GenScript (Piscataway).^12^ The amino terminus serotypic peptides for each GAS strain are defined^9^ and synthesized at China Peptides Co., Ltd. (Jiangsu, China).

### Vaccination and sequential GAS infection protocol

BALB/c mice were either immunized s.c. on days 0, 21, and 28 with 30 μg of J8-DT/Alum or left untreated. Serum samples were taken prior to and 1 week after each immunization. Three weeks after the last boost, mice were sequentially infected with various GAS strains, at 1 × 10^6^ CFU/mouse, via the skin as described.^9^ Each infection was followed for up to 3 weeks to confirm the clearance of bacteria from the skin prior to subsequent infection. J8-DT immunization was followed by co-infection with different strains. Age-matched naïve mice were used as challenge controls.

### Sample collection, antibody, and CFU determination

Following vaccination, 6 days after each sequential infection or co-infection, five mice were euthanized to obtain skin samples for CFU quantification. To allow for detection of current and previous GAS infection and different GAS strains following co-infection experiments, specific antibiotic-laced blood agar plates were used. Serum samples were collected at various time points post each infection and serum IgG levels specific for J8, SpyCEP, or N-terminal peptides from the M protein were quantified as described previously.^9^

### Detection of ASCs with ELISPOT

Splenocytes were analyzed at specific time points by ELISPOT as described.^9^ The use of J8 or SpyCEP peptides allowed quantification of specific ASCs.

### Statistical analysis

Data were analyzed using GraphPad PRISM version 6.0 for Macintosh. Statistical analysis was performed using ANOVA with a Tukey or Dunnett’s post hoc method for multiple comparisons. A *p-*value < 0.05 was considered statistically significant.

### Data availability

The authors declare that data supporting the findings of this study are available within the paper and its Supplementary Information files.

## Electronic supplementary material

Supplemental Data
