# Supplementary material for: Skin infection boosts memory B-cells specific for a cryptic vaccine epitope of group A streptococcus and broadens the immune response to enhance vaccine efficacy
Source: NPJ Vaccines. 2018 Apr 26;3:15. doi: 10.1038/s41541-018-0053-9 (PMC5920117; doi:10.1038/s41541-018-0053-9)
Supplement: Supplementary file 1 — Supplemental Data [file 41541_2018_53_MOESM1_ESM.pdf]

Supplementary figure 1

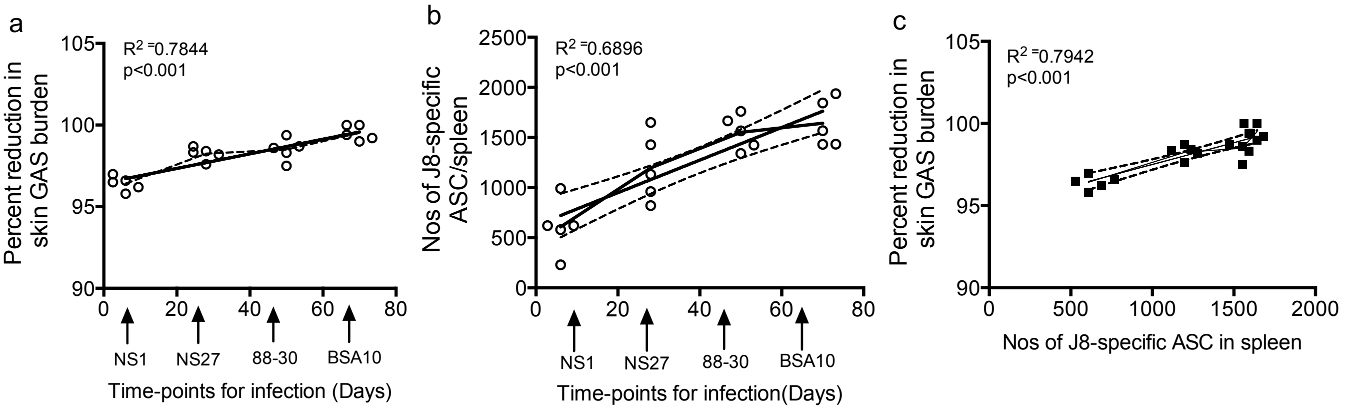

Sup Fig 1 (a). Scattergram and linear regression analysis following sequential GAS infection. BALB/c mice (n=10/group; female, 4-6 week old) were sequentially infected multiple times with NS27 or four different GAS strains NS27, NS1, 88/30 and BSA10 via the skin. At designated time-points post-each infection, mice were culled and J8-specific ASC responses and percent reduction in bacterial burden assessed (Figure 1 B & C). Linear regression analysis was performed to define the relationship between (i) the numbers of infections and percent reduction in skin GAS burden, (ii) number of GAS infection and the number of ASCs and (iii) the and the number of ASCs and percent reduction in GAS burden. The analysis was performed using GraphPad PRISM version 6.00 for Macintosh and is presented as a scattergram.

Supplementary figure 2

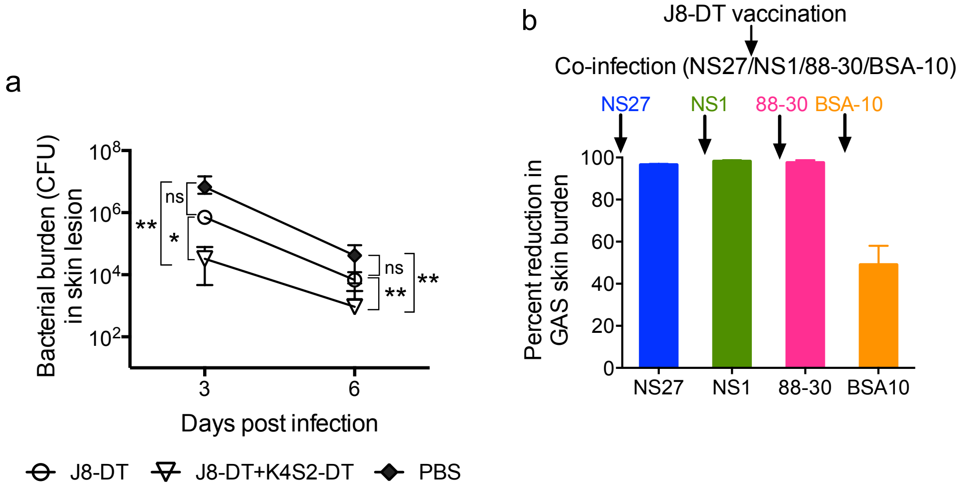

Sup Fig 2. (a) Protective efficacy of J8-DT and J8-DT CombiVax in protection against hypervirulent BSA10 GAS. Cohorts of BALB/c mice (n=10/group) were immunised subcutaneously on days 0, 21 and 28 with either J8-DT, J8-DT CombiVax (J8-DT+K4S2-DT) or PBS, all formulated in Alum. K4S2 is an immunogenic synthetic fragment of SpyCEP [15]. Two weeks after the last immunisation mice were challenged with BSA10 GAS via the skin route of infection. On day 3 and 6 post infection, 5 mice/group were sacrificed and skin samples collected to determine bacterial burden. The mean ±SEM counts in skin are shown. Statistical analysis was performed using one-way ANOVA with Tukey's multiple comparison tests to compare each group with every other group at each time point. *$p<0.05$; **$p<0.01$. **(b) Protective efficacy of J8-DT following a single co-infection with various GAS strains.** Cohorts of BALB/c mice were immunised subcutaneously with J8-DT/Alum. Two weeks post last immunisation mice were challenged via the skin route of infection using a cocktail of GAS strains including NS27, NS1, 88/30 and BSA10. On day 6 post-infection, mice were sacrificed and skin samples collected to determine bacterial burden. Percent reduction in skin bacterial burden in comparison to the corresponding naïve challenge controls was calculated and is shown as mean ±SEM.

Supplementary figure 3

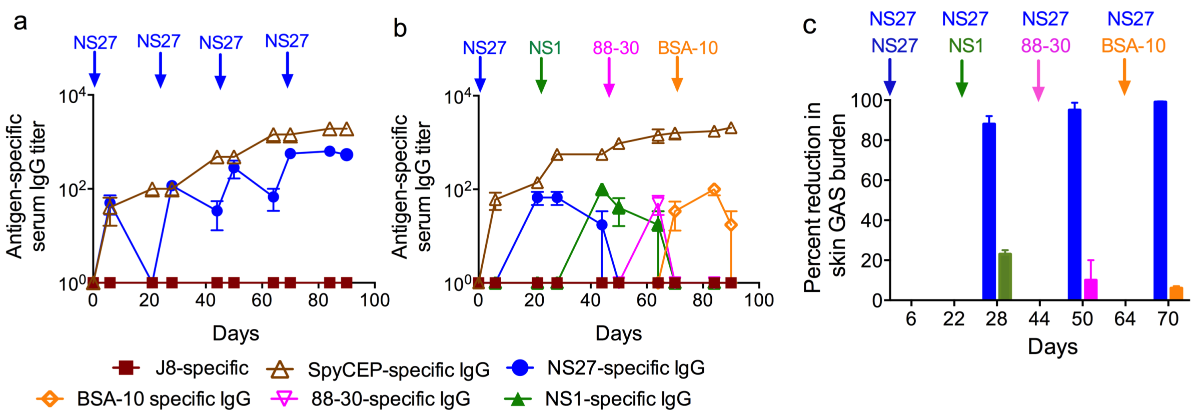

Sup Fig 3. (a-b) Immune responses following sequential GAS infections. BALB/c mice (n=20/group) were sequentially infected via the skin with the same NS27 (a) or four different GAS strains NS27, NS1, 88-30 and BSA10 (b). Each infection was 3-weeks apart. On days 6 and 21 post each infection, sera were collected and M protein N-terminal-specific, J8-specific or SpyCEP- specific IgG were measured via ELISA. Sera from the naïve-uninfected mice were used as a control in ELISA. Data are mean ±SEM. **(c) Protective immunity following sequential skin infections with the same or multiple GAS strains.** On day 6 post each infection, 5 mice from each cohort were sacrificed and skin samples collected to determine bacterial burden. Naïve BALB/c mice were used as a challenge control at each time-point. Percent reduction in skin bacterial burden was calculated by taking into account the corresponding naïve challenge controls and is shown as mean ±SEM.
